# Correlation between Serum RANTES Levels and the Severity of Parkinson's Disease

**DOI:** 10.1155/2014/208408

**Published:** 2014-12-22

**Authors:** Peng Tang, Li Chong, Xiaoqing Li, Yue Liu, Peng Liu, Chen Hou, Rui Li

**Affiliations:** Department of Geriatric Neurology, Shaanxi Provincial People's Hospital, No. 256 Youyi Road, Xi'an 710068, China

## Abstract

Inflammatory mediators may reflect a role of systemic inflammation in the neurodegenerative process of Parkinson's disease (PD). Interleukin-6 (IL-6) and chemokine ligand 5 (CCL5), also known as RANTES (regulated on activation, normal T cell expressed and secreted), have been implicated in neurodegenerative diseases including PD. Serum levels of RANTES and IL-6 of 78 consecutive PD patients and age-matched 80 controls were measured. Patients with PD had higher RANTES and IL-6 levels compared with the controls. We found that serum RANTES levels strongly correlated with Hoehn-Yahr score and disease duration in PD patients. This study indicated that patients with PD have an on-going systemic inflammatory profile where the elevated peripheral production of RANTES may play a role in the neurodegenerative process.

## 1. Introduction

Parkinson's disease (PD), a common neurodegenerative disease among the elderly, is characterized by resting tremor, slowness of movement, rigidity, and postural instability. Although the etiology of PD still remains under investigation, accumulating data have revealed that the neuroinflammatory mechanisms contribute to the neurodegenerative process of PD [1, 2]. In a PD-affected brain, a sustained activation of microglial cells in the substantia nigra indicates an overactivation of neuroinflammation in PD progression [2–4]. Interleukin-6 (IL-6) is one of the primary proinflammatory chemokines involved in the augmentation of inflammation. A study reported that circulating level of IL-6 was elevated in the PD patients on average 4.3 years before the diagnosis, suggesting that the inflammation state was not limited in the local central region but expanded to the peripheral ones [5]. Another proinflammatory chemokine RANTES (known as regulated on activation, normal T cell expressed and secreted) has been implicated in the recruitment of immune cells, fundamental regulation of immunoreactions, and hence the maintenance of inflammatory states [6]. Evidences have shown that RANTES and its receptor CCR5 play a role in a wide array of pathological conditions with neurodegenerative diseases such as PD, Alzheimer's disease (AD), multiple sclerosis, stroke, and HIV-associated dementia [7–9]. However, the systemic profiles of RANTES and IL-6 in PD patients have not been fully established. Hence, we investigated the relationship between RANTES, IL-6 levels, and the severity of the disease in PD patients, intending to describe the peripheral inflammatory profiles of PD patients.

## 2. Experimental Procedure

### 2.1. Patients and Controls

PD patients (*n* = 78) who fulfilled the UK Parkinson's Disease Society Brain Bank Clinical Diagnostic Criteria were registered from the clinic of Shaanxi Provincial People's Hospital from October 2008 to May 2011 in a consecutive way. All included subjects with a clinical history of active infectious or systemic inflammatory disease or patients taking corticosteroids were excluded. PD patients were evaluated with UPDRS and Hoehn-Yahr score during their “off” period. The information about antiparkinsonian medications was also analyzed. The approval from the Ethics Committee of Shaanxi Provincial People's Hospital was obtained and written informed consent was provided by all of the participants before enrollment into the study.

Age and sex matched volunteers (*n* = 80) were recruited from Physical Examination Center of Shaanxi Provincial People's Hospital, which was defined as the control group of study. All people from this group were healthy and showed no parkinsonian symptoms.

### 2.2. RANTES and IL-6 Measurement

Blood samples (5 mL) from patients and controls were collected in the morning (8:00–10:00) after an overnight fast with a serum separator tube and clot for 30 minutes at room temperature before centrifugation for 15 minutes at 1000 ×g. Then the serum was collected and the samples were stored at −80°C for later analysis. The Quantikine human CCL5/RANTES and IL-6 from R&D systems (Minneapolis, MN, USA) were, respectively, used for measuring RANTES and IL-6 in serum of all patients and controls. The optical density was determined at 450 nm and 570 nm.

### 2.3. Statistical Analysis

Results are expressed as the mean ± SE unless otherwise specified. Student's *t*-test was used to assess group difference. Spearman's correlation coefficient was applied to test all correlations. Results with probability less than 5% (*P* < 0.05) were considered statistically significant. Statistical analyses were performed using SPSS for Windows, version 13.0 (SPSS Inc., Chicago, Illinois, USA).

## 3. Results

**Figure 1:**
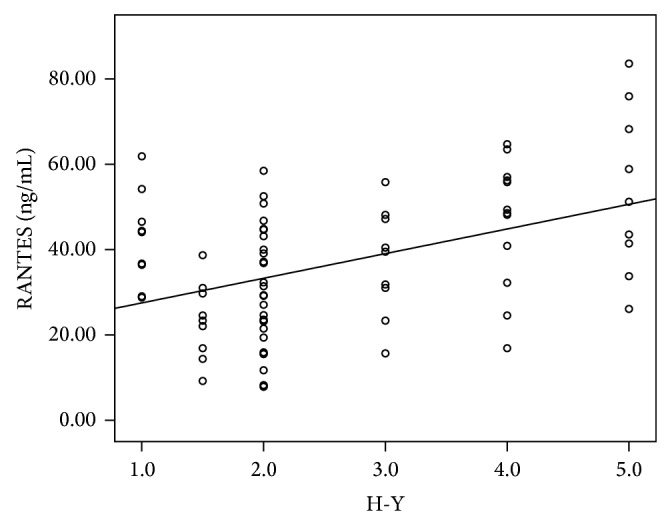
Significant positive correlation between RANTES and H-Y scale in PD patients (*n* = 78, *r* = 0.362, *P* = 0.001).

**Figure 2:**
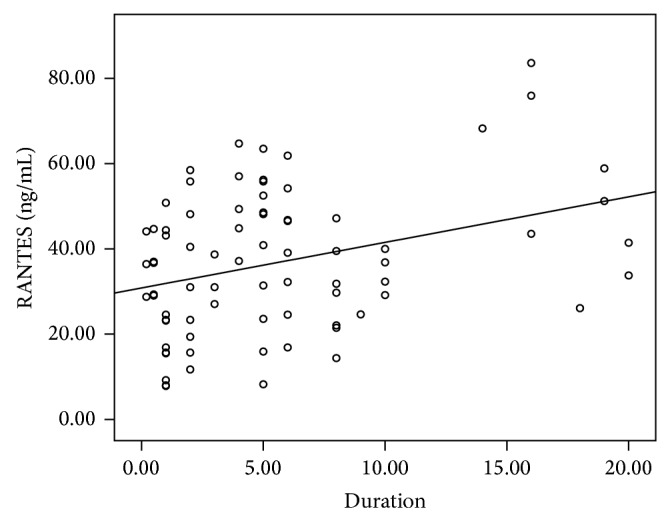
Significant positive correlation between RANTES and disease duration in PD patients (*n* = 78, *r* = 0.275, *P* = 0.015).

Clinical and demographical characteristics of patients and controls are summarized in Table 1 and Figures 1-2. There was no significant difference between patients and controls with respect to the age, sex, smoking, and alcohol habits. The PD group showed significantly increased RANTES and IL-6 levels compared to the controls (*P* = 0.013 and *P* < 0.001, resp.). The serum RANTES and IL-6 levels showed no significant difference between subgroups of PD patients treated and not treated with antiparkinson drugs (Table 2). We found RANTES serum levels presented a correlation with Hoehn-Yahr score, which scaled the severity of PD (*n* = 78, *r* = 0.362, *P* = 0.001). There was a significant positive correlation between RANTES and disease duration in PD patients (*n* = 78, *r* = 0.275, *P* = 0.015). However, there were no associations between RANTES serum levels and UPDRS I, UPDRS II, and UPDRS III (data not shown). Unexpectedly, IL-6 levels were not correlated with Hoehn-Yahr scores, disease duration, and UPDRS I, UPDRS II, and UPDRS III (data not shown).

## 4. Discussion

Several lines of studies have demonstrated that some peripheral inflammatory surrogates such as TNF-alpha [10, 11], IL-6 [12, 13], and IL-10 [14] as well as RANTES are elevated in PD patients. Our study has confirmed that RANTES and IL-6 serum levels increased in PD patients compared with sex- and age-matched subjects. To extend the finding, we further investigated the association between RANTES and PD severity. We found that there was a strong correlation between RANTES levels and Hoehn-Yahr scores as well as disease duration in PD patients, showing that serum concentration of RANTES increased in a stepwise fashion with Hoehn-Yahr scores, reflecting that the peripheral RANTES level may indicate the severity of PD symptoms. In this regard, RANTES may be served as a surrogate biomarker in the evaluation of the PD severity. Since the deterioration rate is heterogeneous among PD patients, the finding of a biomarker relevant to the severity of PD may be beneficial to the diagnosis and monitoring of the disease in a clinical context.

Meanwhile, our data support the notion that a chronically systemic inflammation state may play a role in the progression of PD. Multiple studies have indicated that there is a cross talk between systematic inflammation and neuronal damage in neurodegenerative diseases. For instance, in primary neurodegenerative diseases such as PD and AD, the initial event may be a nonimmune-mediated injury within the central nervous system. However, accompanied with the local resident microglial activation, peripheral immune cells such as macrophages and monocytes as well as lymphocytes were recruited into the injured site, under certain conditions, and elicited a secondary inflammatory reaction, which is believed to contribute to the neuronal damage, where a list of key proinflammatory mediators, including IL-6 and RANTES, plays a profound role [1, 2]. Thus, the inflammation profile in PD appeared to be not merely a local substantia nigra involvement, but a systematic inflammation disorder.

The correlation between the elevated serum RANTES and the severity of the disease may be attributed to the following possibilities. One possibility could be due to the leakage of RANTES from the inflammatory reaction region in the CNS, since the sustained neuroinflammation-induced vicious cycle may facilitate the neuronal damage as in PD progression, in which a host of proinflammatory factors such as IL-6, TNF-*α*, and chemokines was overexpressed in the specific site in the brain and these factors could leak through blood-brain barriers, thus causing an elevated RANTES level in the peripheral blood. It does not seem to be reasonable, however, because the concentration of RANTES in the cerebral spinal fluid (CSF) of the patients with ALS is far lower (under 1 ng/mL) than that in the peripheral blood (over 90 ng/mL) [15]. Another explanation for the elevated serum RANTES level could be the increased production of RANTES in the blood itself. It was reported that the RANTES concentration in culture supernatants of the total peripheral blood mononuclear cells from PD patients was 1.7 times higher than that in healthy controls when challenged with LPS by Reale et al. [16]. Meanwhile, platelets can bind and internalize IgG-coated particles and then become activated and capable of releasing RANTES [17]. Moreover, platelet antigen-1a antibody was capable of inducing the release of RANTES from human platelets* in vitro* [18]. Thus the correlation between RANTES serum levels and disease severity reflects an important role of peripheral inflammatory states in PD progression.

The reasons for the lack of association between IL-6 levels and the severity of the disease are unclear. First, we could not exclude the possibility of chance or unmeasured confounding as alternative explanations for this. Furthermore, single point measurements of plasma biomarkers of inflammation are subject to day-to-day variations. In addition, the small sample size limited our statistical power in the analyses. Finally, differences in clearance of IL-6 and RANTES could partially explain the difference.

A previous study found a significant correlation between RANTES levels and UPDRS III score [19]; however, in our investigation we failed to address a correlation between RANTES levels and UPDRS III scores. The discrepancy between the two results may result from the difference of average age of PD patients enrolled in the studies. The average age of the patients was younger in the previous study than that in our study (67.5 versus 76.3 years). Meanwhile, it was considered that rigidity and bradykinesia scores were higher among old-aged PD patients than that in middle-aged ones in UPDRS assessment [20]. Further, it was believed that UPDRS III assessment exhibited more sensitivity in tremor-dominant patients than in rigidity- and bradykinesia-dominant subjects [21]. Therefore, the confounding factor of average age may impact the correlation between RANTES levels and UPDRS III score. In this regard, our study suggested a more reliable and sensitive assessment instrument dedicated to the rigidity- and bradykinesia-dominant PD patients was needed.

The present study had some limitations. Although serum RANTES levels were correlated with Hoehn-Yahr score and disease duration in PD patients, the relatively low value of *r* presented a weak positive linear relationship. Thus, more powerful inflammatory biomarkers reflecting the severity of PD should be investigated. In addition, PD is a heterogeneous disease where genetic and environmental causes are involved. It would be plausible to consider that inflammation process may be influenced by both environmental conditions and genetic backgrounds. Thus, environmental exposition and genetic analysis should be incorporated in the following study.

## 5. Conclusion

This study demonstrates that increased serum RANTES levels in PD patients were associated with the severity of PD, which points to importance of the systemic inflammation in PD progression. Future studies should be conducted to elucidate the exact origination of the elevated RANTES and the contribution of peripheral RANTES in PD progression.

## Figures and Tables

**Table 1:** Characteristics of subjects.

Subjects	PD	CTRL
*N*	78	80
M/F	60/18	59/21
Smoking	10	18
Alcohol habits	12	16
Age (years)	76.3 ± 5.0	75.4 ± 4.4
H-Y scale	2.60 ± 1.26	
Duration (years)	5.51 ± 5.21	
UPDRS I	2.85 ± 2.12	
UPDRS II	13.88 ± 7.108	
UPDRS III	27.15 ± 12.42	
RANTES (ng/mL)	36.72 ± 16.61^*^	31.13 ± 10.65
IL-6 (pg/mL)	4.67 ± 3.64^#^	2.66 ± 1.91

Data are presented as mean ± SD. PD: Parkinson's disease; CTRL: control group; H-Y scale: Hoehn-Yahr scale; UPDRS: Unified Parkinson's Disease Rating Scale; RANTES: regulated on activation, normal T cell expressed and secreted; IL-6: interleukin-6.

^*^ *P* = 0.013 and ^#^ *P* < 0.001 versus CTRL (Student's *t*-test).

**Table 2:** Characteristics of untreated and treated subgroups of PD patients.

Subjects	Untreated	L-Dopa	Agonist^*^	*P*
*N*	21	36	21	
M/F	17/4	27/9	16/5	NS
Age (years)	75.7 ± 5.4	77.2 ± 4.9	75.3 ± 4.6	NS
Duration (years)	3.22 ± 5.13	7.03 ± 4.48	5.21 ± 5.76	0.025
RANTES (ng/mL)	38.28 ± 19.28	33.0 ± 14.51	41.54 ± 16.41	NS
IL-6 (pg/mL)	5.20 ± 3.20	4.16 ± 3.99	5.01 ± 3.46	NS

Data are presented as mean ± SD. PD: Parkinson's disease; RANTES: regulated on activation, normal T cell expressed and secreted; IL-6: interleukin-6; NS: not significant.

^*^Combination of agonist with L-dopa *n* = 18, agonist monotherapy *n* = 3.
